# Spatial–temporal changes of landscape and habitat quality in typical ecologically fragile areas of western China over the past 40 years: A case study of the Ningxia Hui Autonomous Region

**DOI:** 10.1002/ece3.10847

**Published:** 2024-01-22

**Authors:** Ding Wang, Haiguang Hao, Hao Liu, Lihui Sun, Yuyang Li

**Affiliations:** ^1^ Chinese Research Academy of Environmental Sciences Beijing China; ^2^ China National Environmental Monitoring Centre Beijing China

**Keywords:** habitat quality, InVEST model, landscape pattern index, land use change, Ningxia Hui Autonomous Region

## Abstract

In this paper, we use the InVEST model and five periods of land use data from 1980 to 2020 to assess the habitat quality of the Ningxia Hui Autonomous Region in Western China, which has characteristics of a typical fragile ecosystem. We further analyse the spatial and temporal characteristics of habitat quality evolution and its relationship with land use and landscape pattern indices to explore the close relationship between regional habitat quality changes and human natural resource conservation and utilization. The research results show that the overall habitat quality of Ningxia Hui Autonomous Region was stable and at a moderate level (0.57–0.60) during the 40 years from 1980 to 2020; Habitat patches (2020) with low (24.89%), high (22.45%) and very high (29.81%) quality occupy a larger proportion of the area, followed by very low (13.31%) and moderate levels (9.54%). Over the past 40 years, there have been 275 sample sites in Ningxia where habitat quality has deteriorated, 1593 sample sites where the habitat quality has remained stable, and 184 sample sites where the habitat quality has increased. From 1980 to 2020, the Mean Patch Area of landscape types in Ningxia decreased by 25.9 hm^2^. The Patch Density increased by 0.06 /hm^2^. The Largest Patch Index decreased by 15.69%. The Edge Density increased by 2.5 m/hm^2^. The Contagion Index decreased by 2.99%. The Area‐Weighted Mean Patch Fractal Dimension remained basically unchanged (0.01). The Landscape Shape Index showed a trend of first increasing and then decreasing, increasing by 13.94. The Area‐Weighted Mean Shape Index has been reduced by 9.45. The Shannon Diversity Index and Shannon Evenness Index both show an increasing trend, but the amplitude is relatively small, with 0.09 and 0.04, respectively. There was a significant spatial aggregation of high and low habitat quality in Ningxia, with high values usually distributed in the northern and southern areas with good natural conditions and low values distributed in areas with frequent human activities and poor natural conditions. The decrease in habitat quality in Ningxia was mainly due to the expansion of cultivated land and construction land, the increase in landscape fragmentation and the resulting decrease in connectivity. On the other hand, due to the implementation of ecological protection measures, such as the project of returning farmland to pasture and grass to forest, the quality of habitats in Ningxia increased. The conclusions of this study support the idea that the conservation of habitat quality in ecologically fragile areas should fully preserve the original natural habitats and reduce the interference of human activities to increase the habitat suitability of the landscape and the habitat connectivity between patches. At the same time, targeted ecological protection policies should be developed to restore the areas where the habitat quality has been damaged and ultimately maintain the stability of biodiversity and ecosystems in ecologically fragile areas. Meanwhile, for ecologically fragile areas with similar ecological characteristics to those of Ningxia, our research supports the idea of increasing the protection of the stability of the original habitats, increasing the proportion of ecological restoration projects, financial investment and seeking cooperation with local community managers and residents will help to improve the quality of the regional habitats and the enrichment of the biodiversity, and ultimately promote the harmonious coexistence of human beings and nature in the modernized sense of the word.

## Graphical abstract

This study explored the relationship between habitat quality, land use and landscape patterns in ecologically fragile regions and obtained interesting and meaningful results, which were finally presented to peers and readers in the form of charts.

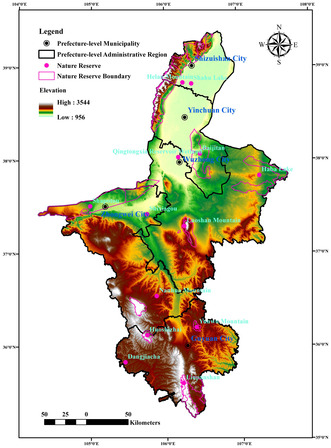

## 1 INTRODUCTION

Ecologically fragile areas are usually located at the junction of different types of ecosystems in excessive areas; their systems are weakly resistant to disturbance, and they are prone to ecological degradation (Yu & Lu, 2011). Ecologically fragile areas are often characterized relatively low socioeconomic levels, strong dependence on natural resources for economic development and obvious pressure on the ecological environment from development and construction activities, and these factors can easily lead to a decrease in regional ecosystem stability (Crabtree & Bayfield, 1998). It is important to evaluate ecosystem functions in ecologically fragile areas and grasp their evolutionary characteristics to ensure national and regional ecological security.

Habitat quality is used to evaluate the ability of an ecosystem to provide suitable living conditions for individuals, populations or communities, and its ability is closely related to the magnitude of ecosystem services, which is an important indicator of ecosystem service functions (Battin, 2004; Kuussaari et al., 2009; Mengist et al., 2021). Habitats provide various resources, that individuals or populations need to survive and thrive, such as food and habitat (Ward et al., 2020; Yan et al., 2021), and their quality directly affects population growth and the stability of biodiversity (Beerens et al., 2015; Michel et al., 2022). Climate change, urban expansion and environmental pollution occur frequently worldwide, leading to species habitat fragmentation and a dramatic decline in biodiversity. Habitat quality has gradually become a prominent topic of research in the field of ecology in recent years (Airoldi & Beck, 2007; Chase et al., 2020; Hale & Swearer, 2016).

The main research perspectives on habitat quality are as follows: regional habitat quality evaluation, the study of habitat quality evolution characteristics and influencing factors, habitat quality future change scenario simulation and driving forces, species‐specific habitat quality evaluation and regional habitat quality restoration pathway measures (Demerdzhiev et al., 2022; Hamere et al., 2021; Li et al., 2022). Assessments of regional scale habitat are based on the advantages and disadvantages of food, habitat, space and other conditions of biological populations in the region and reflect the quality of the overall regional ecosystem and examine whether ecological security can be guaranteed (Shekoufeh et al., 2020). Habitat quality research methods are divided into three types: field survey methods, qualitative methods based on evaluation systems and quantitative methods using model calculations. Ecological assessment models have the characteristics of modelling, spatialization, quantification and refinement that fully combine the advantages of 3S technology and modelling, are applicable on multiple scales and have obvious advantages in evaluating the current status of habitat quality and predicting future habitat distribution (McGarigal et al., 2016). Available quantitative assessment models include the IDSIRI biodiversity module (Shanthala‐Devi et al., 2016), the HIS model of habitat suitability (Wang et al., 2009), the SolVES model (Wang et al., 2016), the InVEST model (Leh et al., 2013) and the MaxEnt model (Merow et al., 2013). The InVEST model is widely used for habitat quality assessment due to the ease of data acquisition, better system construction, simplicity of operation and visualization features and has been more frequently applied internationally (Mushet et al., 2014).

Land use is one of the most direct human surface activities and has caused fragmentation, degradation and even loss of biological habitats as the pattern, depth and intensity of urban and agricultural land expansion has increased. Land use is a fundamental type of human use of natural resources, the results of which affect the ecosystem services obtained by humans and also significantly impact natural ecosystems and landscape patterns (Bateman et al., 2013). Landscape patterns consist of landscape elements of different sizes, shapes and arrangements, and landscape types of different sizes, shapes and densities differ greatly in their roles in maintaining biodiversity, protecting species, improving overall structure and function and promoting natural succession of landscape structures as well as in their resistance and resilience to external disturbances (Baguette et al., 2013; Fahrig, 2013). Landscape patterns can profoundly influence various ecological processes, patch size and connectivity, thus impacting the abundance and distribution of species within the landscape and the survival and resistance of populations to disturbance (Haddad et al., 2015); for example, landscape fragmentation is an important factor leading to habitat fragmentation. Coupling habitat quality evaluation with landscape patterns and quantitatively analysing the characteristic indices of landscape spatial patterns are beneficial to discern the processes and mechanisms of habitat quality changes (Huang et al., 2020; Rahimi et al., 2020; Xia et al., 2018) and summarize the regional ecological environment status from a macroscopic perspective (Fletcher et al., 2018; Tscharntke et al., 2012).

Ningxia is a typical ecologically fragile region in northwestern China. Because it is located in an arid and semi‐arid zone with a single ecosystem structure, scarce and uneven spatial and temporal distribution of precipitation and high evapotranspiration, water resources in Ningxia are extremely strained and at the same time, drought, sandstorms and soil erosion are prominent, and the level of biodiversity faces serious challenges. The overall pressure on ecosystem protection in Ningxia is high, and its biodiversity protection and ecological environment construction are the guarantee for the middle and lower reaches of ecological environment management and sustainable economic and social development. Studies addressing the ecological environment and ecosystem services of Ningxia have been divided into two main areas, one of which considers the ecological status of Ningxia in the region and even in the country, often combining the locational elements of the Yellow River Basin, the Loess Plateau, the arid and semi‐arid areas and the agricultural and pastoral intertwining zones, and the second of which focuses on the ecosystem processes and the socio‐economic drivers within Ningxia. It can be broadly categorized into the northern Yellow River irrigation economic development zone, the central soil erosion and wind and sand control zone and the southern water conservation zone to conduct research on ecosystem service functions such as water conservation, soil conservation, habitat maintenance, wind and sand control and carbon storage. Currently, research on ecosystem services in Ningxia is gradually shifting from land use‐driven ecosystem service changes to new cultural tourism services, multi‐scenario simulation of driving factors, value assessment and realization mechanisms and analysis of nature‐socio‐economic coordination, etc.

The Ningxia Hui Autonomous Region, located in western China, is an important ecological barrier in the northwest of China with a relatively fragile and sensitive ecological environment (Cadavid Restrepo et al., 2017; Li & Ren, 2007; Wang et al., 2014; Wu et al., 2020; Zhou et al., 2016). In this study, we examined land use data from five periods: 1980, 1990, 2000, 2010 and 2020. We analysed the spatial and temporal evolution characteristics of habitat quality in Ningxia over the past 40 years using Fragstats 4.2 software, the InVEST model and the ArcGIS platform and explored the relationship between habitat quality, land use, and landscape pattern. The main scientific problems addressed in this study are as follows: (1) the characteristics and changes of land use and landscape patterns in Ningxia in the last 40 years, (2) the spatial and temporal status and change characteristics of habitat quality in Ningxia in the last 40 years and (3) response of habitat quality to land use and landscape pattern changes in Ningxia. The main novelties and innovations include (1) this study used a 40‐year time scale, which is long enough to have a longer understanding and comparison of land use, landscape pattern and habitat quality in Ningxia, and the difference is larger before and after 2000, which is a big advantage for us to look for the reasons for the changes in habitat quality and biodiversity level; (2) the habitat quality assessment model used in this study can analyse the habitat condition on a macro scale more accurately and combined with the changes in land use and landscape pattern in Ningxia during the 40 years, it can give its response and correlation on a macro scale, and at the same time, Ningxia is located in a typical ecologically fragile area, and the results of this study are of great importance for the maintenance of habitats and the protection of biodiversity in ecologically fragile areas similar to the ecological characteristics of Ningxia.

## 2 MATERIALS AND METHODS

### 2.1 Study area

**FIGURE 1:**
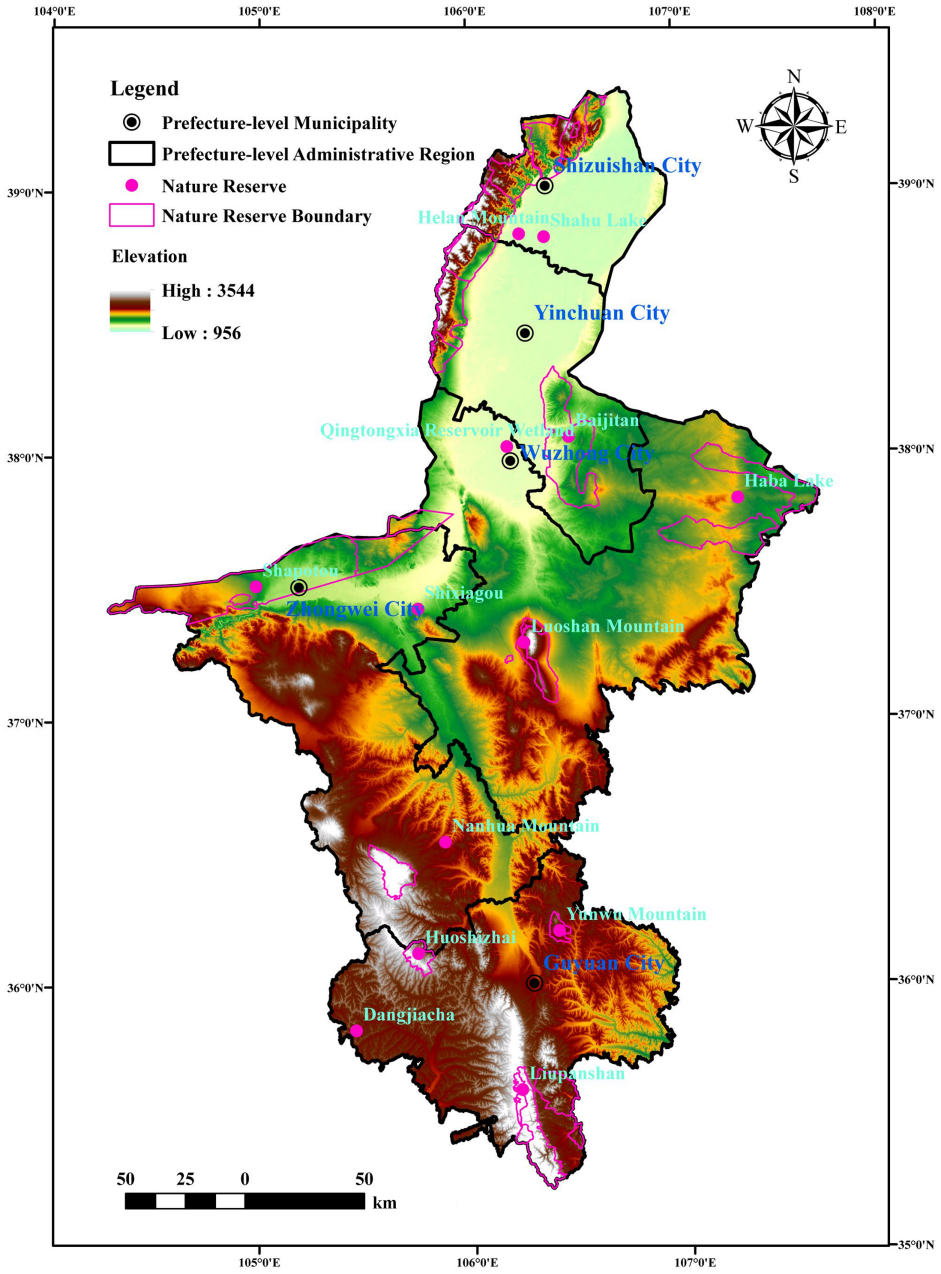
Topography, administrative divisions and nature reserves distribution diagram in Ningxia Hui Autonomous Region.

The Ningxia Hui Autonomous Region (104°17′–107°39′ E, 35°14′–39°23′ N) is located in northwestern China, in the transition zone between the Loess Plateau and Inner Mongolia Plateau, in the transition zone of arid and semiarid regions and in the interlocking area of agriculture and animal husbandry. It is an important part of China's Loess Plateau Sichuan‐Yunnan ecological barrier and northern sand control belt (Figure 1). Ningxia has a typical continental semihumid and semiarid climate, with an average annual temperature of 6–10°C, an average annual precipitation of 220 mm, a rainy season concentrated between June and September, and more than 32:00 h of sunshine. The average elevation of the entire Ningxia region is above 1000 m, divided into three major sections: the southern hilly mountainous area, the central arid area and the northern yellow irrigation area (with landscape types such as Liupan Mountain, Loess Hills, central mountainous hilly basin, Lingyan Terrace, Ningxia Plain and Helan Mountain from south to north). The northern yellow irrigation area has superior natural conditions, a good development foundation, population and industry concentrations, and a high level of land use and urbanization. The central arid area has a fragile natural ecological environment, relatively lagging socioeconomic development, prominent land desertification and salinization. The southern mountainous area belongs to the national poverty area, with a regressing productivity level. The vegetation from south to north is forest grassland, dry grassland, desert grassland and grassland desert, and the soil is mostly black kiln soil and grey calcium soil. Species diversity and ecosystem diversity are relatively small; the forest cover is only 16.9%, with singular vegetation composition and a relatively poor abundance of biological species. Due to its proximity to deserts such as Tengri, Ulanbu and Mao Wu Su and the Loess Plateau, the area in Ningxia affected by wind, sand, drought and soil erosion accounts for 23.63% of the total area of the region and 53.68% of the total area of desertified land. Natural grassland in Ningxia accounts for 47% of the total area, but due to long‐term overloaded grazing and reclamation damage, the proportion of the regions with moderate or severe degradation has reached 77.6%. In recent years, the ecological environment level of Ningxia has changed from a ‘highly fragile area’ to a ‘very highly fragile area’.

### 2.2 Research methodology

#### 2.2.1 Dynamic degree of land use change and landscape pattern index

In this paper, the single land use type dynamic attitude was used to reflect the degree of land use change of a certain type of land use in Ningxia from 1980 to 2020, and the calculation formula is as follows.

(1) $$K=\frac{U_{a}-U_{b}}{U_{a}}\times \frac{1}{T}\times 100\%,$$

where *K* is the dynamic attitude of a certain land use type during the study period, %; *U*_*a*_ and *U*_*b*_ are the proportion of the area of a certain land use type at the beginning and end of the study period, %, respectively, and *T* is the study time, *a*. The composite land use type dynamic attitude of the study area is the sum of the absolute values of *K* for each land use type, %.

The landscape pattern index reflects the quantity and area of the landscape and its change characteristics, reflects landscape heterogeneity and ecological processes, explains landscape structure and dynamics and, to a certain extent, can reflect the quantity maintenance and spatial configuration of habitats and indicate habitat quality characteristics. The density, area edge, shape and diversity indicators were selected to characterize the landscape pattern changes in Ningxia, Mean Patch Area (MPA), Patch Density (PD), Largest Patch Index (LPI), Edge Density (ED), Contagion Index (CONTAG), Area‐Weighted Mean Patch Fractal Dimension (AWMPFD), Landscape Shape Index (LSI), Area‐Weighted Mean Shape Index (AWMSI), Shannon's Diversity Index (SHDI), Shannon's Evenness Index (SHEI) and a total of 10 indices (Hu et al., 2022; Leitão & Ahern, 2002; Zhu et al., 2020) (Table 1).

**TABLE 1:** Landscape metrics.

Landscape index	Unit	Category	Ecological meaning
Mean Patch Area	hm^2^	Density indicators	Indicates the number and area of patches within the landscape, which can reflect the degree of habitat crowding and has a strong positive correlation with the degree of landscape fragmentation
Patch Density	/hm^2^		
Largest Patch Index	%	Edge indicator	Reflects the degree of edge mosaic of different patches, which affects species migration and leapfrogging, and has a large impact on species dispersal
Edge Density	m/hm^2^		
Contagion Index	%		
Area‐Weighted Mean Patch Fractal Dimension	/	Shape indicator	Reflects the complexity of habitat patterns, the complexity of patch shape has an influence on many ecological processes and is closely related to human activity disturbance
Landscape Shape Index	/		
Area‐Weighted Mean Shape Index	/		
Shannon's Diversity Index	/	Diversity indicators	Reflects the diversity of habitat patches in a region and has an impact on the selection of habitats for many species
Shannon's Evenness Index	/		

#### 2.2.2 Habitat quality assessment

The habitat quality module of the InVEST model assesses habitat degradation and habitat quality indices for each land use based on information on threats to biodiversity with the help of quantitative ecological parameters and land use patterns. The habitat quality index was calculated as follows:

(2) $$Q_{\mathrm{xj}}=H_{j}\left[1-\left(\frac{D_{\mathrm{xj}}^{z}}{D_{\mathrm{xj}}^{z}+k^{z}}\right)\right],$$

where *Q*_*xj*_ is the habitat quality of raster *x* in land use type *j*; *k* is the half‐saturation parameter, whose value is half of the resolution of the raster data in the study area and is generally 1/2 of the maximum value of habitat degradation; *H*_*j*_ is the habitat suitability of land use type *j*, whose value is usually 0–1; *z* is the normalization constant, which is usually set to 2.5 and *D*_*xj*_ is the level of stress to which raster *x* of land use type *j* is subjected, that is, the degree of habitat degradation. The degree of habitat degradation is the intensity of habitat disturbance by threat sources and is calculated as follows:

(3) $$D_{\mathrm{xj}}=\sum_{r=1}^{R}\sum_{y=1}^{y_{r}}\left(\frac{\omega_{r}}{\sum_{r=1}^{R}\omega_{r}}\right)r_{y}i_{\mathrm{rxy}}\beta_{x}S_{\mathrm{jr}},$$

(4) $$i_{\mathrm{rxy}}=1-\left(\frac{d_{\mathrm{xy}}}{d_{\text{rmax}}}\right)\left(\text{Linear Decay}\right),$$

(5) $$i_{\mathrm{rxy}}=\exp\left(\frac{-2.99d_{\mathrm{xy}}}{d_{\text{rmax}}}\right)\left(\text{Linear Decay}\right),$$

where *D*_*xj*_ is the degree of habitat degradation; *R* is the number of stressors; *y* is the number of grids in the raster layer of stressor *r*; *y*_*r*_ is the number of grids occupied by stressors; *ω*_*r*_ is the stressor weight; *r*_*y*_ is the stressor value of raster *y*; *β*_*x*_ is the reachability level of raster *x*, which is not considered in this study; *S*_*jr*_ is the sensitivity of habitat type *j* to stressor *r*; *i*_*ryx*_ is the stress factor value *r*_*y*_ of raster *y* on the stress level of habitat raster *x*; *d*_*xy*_ is the linear distance between raster *x* and raster *y*; and *d*_*rmax*_ is the maximum stress distance of threat source *r*. The higher the calculated score is, the greater the threat level caused by the threat factor to the habitat and the higher the habitat degradation.

Based on the InVEST model manual and with reference to previous research results on habitat quality in Ningxia and arid and semiarid regions of northwest China (Bao et al., 2015; Lyu et al., 2018), in this study, we used paddy fields, drylands, urban land, rural settlements and other construction land as threat factors and determined the habitat suitability of habitat types and the sensitivity of different habitat types to stress factors (Tables 2 and 3).

**TABLE 2:** Ecological threat sources and their maximum impact distance and weight.

Threat factor	Impact distance/km	Weighting	Spatial decline type
Paddy field	4	0.15	Linear decline
Dryland	3	0.2	Linear decline
Urban land	5	0.3	Exponential decline
Rural settlement	4	0.3	Exponential decline
Other construction	8	0.2	Linear decline

**TABLE 3:** Habitat types and sensitivity of habitat types to each threat.

Type	Habitat suitability	Paddy field	Dryland	Rural settlement	Urban land	Other construction land
Paddy field	0.6	0.3	0.2	0.35	0.5	0.45
Dry land	0.4	0.3	0.2	0.35	0.5	0.4
Wooded land	1	0.8	0.7	0.85	1	0.6
Shrubland	1	0.4	0.3	0.45	0.6	0.4
Open forest land	1	0.85	0.75	0.9	1	0.65
Other wooded land	1	0.9	0.8	0.95	1	0.7
High‐cover grassland	0.85	0.4	0.3	0.45	0.6	0.6
Grassland with medium cover	0.8	0.45	0.35	0.5	0.65	0.7
Low‐cover grassland	0.75	0.5	0.4	0.55	0.7	0.8
River and canal	1	0.7	0.6	0.75	0.9	0.5
Lakes	1	0.7	0.6	0.75	0.9	0.5
Reservoir ponds	1	0.7	0.6	0.75	0.9	0.5
Beachland	0.6	0.75	0.65	0.75	0.95	0.55
Urban land	0	0	0	0.8	0	0
Rural settlements	0	0	0	0	0	0
Other construction land	0	0	0	0	0	0
Untapped land	0	0	0	0	0	0

#### 2.2.3 Spatial autocorrelation and cold/hot spot analysis

In this study, the global Moran's *I* index was used to describe whether habitat quality in the study area has a regional clustering effect, and the local Moran's *I* index was used to reflect the spatial autocorrelation of habitat quality in subregions. The specific calculation formula is as follows:

(6) $${\text{Global Moran}}^{\prime }s\;I=\frac{n\sum_{i=1}^{n}\sum_{j=1}^{n}\omega_{\mathrm{ij}}\left(x_{i}-\bar{x})(x_{j}-\bar{x}\right)}{\sum_{i=1}^{n}{\left(x_{i}-\bar{x}\right)}^{2}\left(\sum_{i}\sum_{j}\omega_{\mathrm{ij}}\right)},$$

(7) $${\text{Local Moran}}^{\prime }s\;I=\frac{\left(x_{i}-\bar{x}\right)}{\sum_{i=1}^{n}{\left(x_{i}-\bar{x}\right)}^{2}}\sum_{j=1}^{n}\omega_{\mathrm{ij}}\left(x_{i}-\bar{x}\right)\;\left(i\neq j\right),$$

(8) $${\text{Local Moran}}^{\prime }s\;I=Z_{i}\sum_{j}\omega_{\mathrm{ij}}Z_{j}\;\left(i\neq j\right),$$

(9) $$\text{LISA}=\frac{\left(x_{i}-\bar{x}\right)}{\sum_{i}\frac{{\left(x_{i}-\bar{x}\right)}^{2}}{n}}\sum_{j}\omega_{\mathrm{ij}}\left(x_{i}-\bar{x}\right)\;\left(i\neq j\right),$$

(10) $$Z=\frac{I-E_{i}}{\sqrt{\mathrm{var}\left(I\right)}},$$

(11) $$G_{i}^{*}=\frac{\sum_{j=1}^{n}\omega_{i,j}x_{j}-X\sum_{j=1}^{n}\omega_{i,j}}{\sqrt[{S}]{\frac{\left[n\sum_{j=1}^{n}\omega_{i,j}^{2}-{\left(\sum_{j=1}^{n}\omega_{i,j}\right)}^{2}\right]}{\left(n-1\right)}}},$$

(12) $$S^{2}=\frac{1}{n}\sum_{i=1}^{n}{\left(x_{i}-\bar{x}\right)}^{2},$$

where *x*_*i*_ and *x*_*j*_ are the values of variable *x* taken on neighbouring cells, *x* is the attribute value of *n* location variables, $\bar{x\;}$ is the mean value of spatial variable attributes, *ω*_*ij*_ is the spatial weight matrix of raster *i* and raster *j*, *S* is the standard deviation of habitat quality and *n* is the total number of rasters.

#### 2.2.4 Data source and processing

In this study, the land use data of Ningxia for a total of five periods in 1980, 1990, 2000, 2010 and 2020 were obtained from the Resource and Environment Science and Data Center of the Chinese Academy of Sciences (https://www.resdc.cn/) (Xu et al., 2020). The land use data were generated based on Landsat TM imagery of the United States and manually interpreted visually with a resolution of 30 m and a kappa accuracy of over 90%, which meets the needs of land use change analysis in this study. The land use data of Ningxia had a total of 22 subcategories, such as paddy fields and drylands. The ArcGIS platform was used to reclassify the land use types into six primary types: (1) cultivated land, (2) forestland, (3) grassland, (4) water, (5) construction land and (6) untapped land. The corresponding secondary types needed for this study were also obtained, which were convenient for extracting threat factor source raster data and other analyses. Habitat quality results calculated by the InVEST model were gridded and attribute connected, the grid image scale was chosen to be 500 m, and sampling points were constructed for spatial autocorrelation analysis and the analysis of coldspots and hotspots. The results of habitat quality, land use and landscape index were counted to sampling points and corresponding land use types using ArcGIS 10.7 multivalue extraction to point tool, and after removing the outliers, a total of 2052 sample points data and habitat quality values and change status of different land use types in Ningxia were obtained and used to analyze the response of habitat quality to land use and landscape pattern. All spatial data within the article were uniformly converted to the WGS 1984 UTM Zone 48N coordinate system using the ArcGIS10.7 tool to facilitate spatial operations.

## 3 RESULTS

### 3.1 Changes in land use and landscape pattern in Ningxia

**FIGURE 2:**
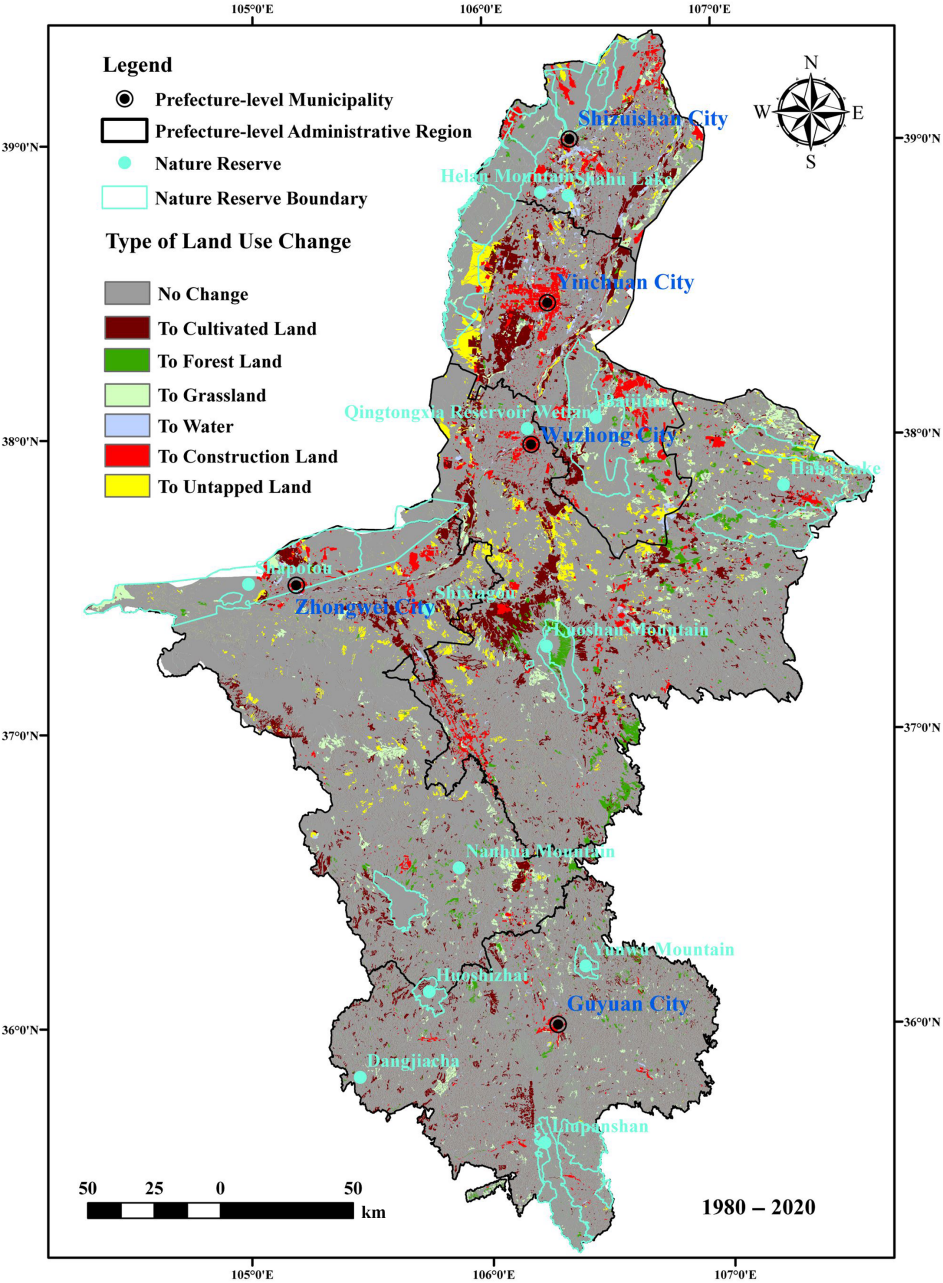
Land use change in Ningxia from 1980 to 2020.

From 1980 to 2020 (Figure 2), cultivated land and grassland were the two types of land use in Ningxia, accounting for a relatively large proportion of 33.30% and 47.61%, respectively, and the proportional size of the ecosystem type area was as follows: grassland > cultivated land > forestland > untapped land > water area. During the 40 years, the land use types that increased in Ningxia were cultivated land (2.74%), forestland (0.96%) and construction land (2.79%), whereas the areas of grassland (−5.36%), water (−0.29%) and untapped land (−0.83%) generally showed a decreasing trend. The proportion of cultivated land and construction land increased more (2.74%, 2.79%), reflecting the steady improvement of Ningxia's socioeconomic level and the demand for more food and land for population growth and urbanization. The proportion of grassland area reached 5.36%, reflecting that Ningxia conducts agro‐industrial development activities mainly using the grassland ecosystem. The area of forestland and construction land increased, whereas the area of cultivated land increased for 20 years and then decreased in the last two decades. From 1980 to 2020, the area of forestland increased and the trend of expanding cultivated land and construction land was clear. The proportion of decrease in grassland in Ningxia in the period from 2000 to 2020 was obviously lower than that from 1980 to 2000, reflecting the remarkable effect of the project of returning farmland to forest and grass in Ningxia. However, the pressure exerted by social and economic construction on the maintenance of the health of the ecosystem was still high.

From 1980 to 2020, the MPA of Ningxia landscape types decreased from 213.93 ha in 1980 to 188.03 ha in 2020; the PD increased from 0.47 to 0.53; the LPI decreased from 37.23 to 21.54; the ED increased 26.7 to 29.2; the CONTAG decreased from 60.11 to 57.12; the AWMPFD remained basically with values of 1.29 and 1.28; the LSI increased from 154.84 in 1980 to 170.78 in 2010 and then slightly decreased to 168.78 in 2020; the AWMSI decreased from 60.68 to 51.23; both SHDI and SHEI exhibited a slightly increasing trend; SHDI increased from 1.23 to 1.32 and SHEI increased from 0.69 to 0.73 (Table 4).

**TABLE 4:** Changes of Ningxia landscape metrics from 1980 to 2020.

Item	Year				
	1980–1990	1990–2000	2000–2010	2010–2020	1980–2020
Mean Patch Area/hm^2^	−0.71	−19.04	−8.23	2.08	−25.9
Patch Density/hm^2^	0	0.05	0.02	−0.01	0.06
Largest Patch Index/%	−0.06	−3.66	−5.98	−5.99	−15.69
Edge Density m/hm^2^	0.14	2	0.57	−0.21	2.5
Contagion Index/%	0.19	−1.05	−1.57	−0.56	−2.99
Area‐Weighted Mean Patch Fractal Dimension	0	0	−0.01	0	−0.01
Landscape Shape Index	0.8	11.9	3.24	−2	13.94
Area‐Weighted Mean Shape Index	0.05	1.71	−11.21	0	−9.45
Shannon's Diversity Index	−0.01	0.03	0.05	0.02	0.09
Shannon's Evenness Index	−0.01	0.02	0.02	0.01	0.04

### 3.2 Spatial–temporal distribution and change of habitat quality in Ningxia

#### 3.2.1 Spatial–temporal distribution and aggregation characteristics

The natural breakpoint method was used to classify the habitat quality level into five levels: very low (0–0.25), low (0.25–0.4), moderate (0.4–0.6), high (0.6–0.75) and very high (0.75–1). The proportional area at each level was also determined. As seen from Table 5, the average habitat quality of Ningxia in 1980, 1990, 2000, 2010 and 2020 was 0.5950, 0.5995, 0.5764, 0.5761 and 0.5733, respectively; these values were classified as moderate. The habitat quality of the largest proportions of the Ningxia area was high, very high and low, followed by very low and moderate levels (Table 5). In 2020, the proportion of high (29.81%) and very high (22.45%) areas in Ningxia combined exceeded half of the total area, followed by areas of very low (24.89%), low (13.31%) and moderate (9.54%) levels.

**TABLE 5:** Average habitat quality and classification ratio of Ningxia from 1980 to 2020 (%).

Item	Year				
	1980	1990	2000	2010	2020
Average level	0.5950	0.5995	0.5764	0.5761	0.5733
Very low level/%	11.43	11.79	11.86	12.92	13.31
Low level/%	23.05	23.21	26.34	25.22	24.89
Moderate level/%	9.03	9.35	10.23	9.78	9.54
High level/%	26.95	26.78	23.47	22.25	22.45
Very high level/%	29.54	28.87	28.09	29.83	29.81

**FIGURE 3:**
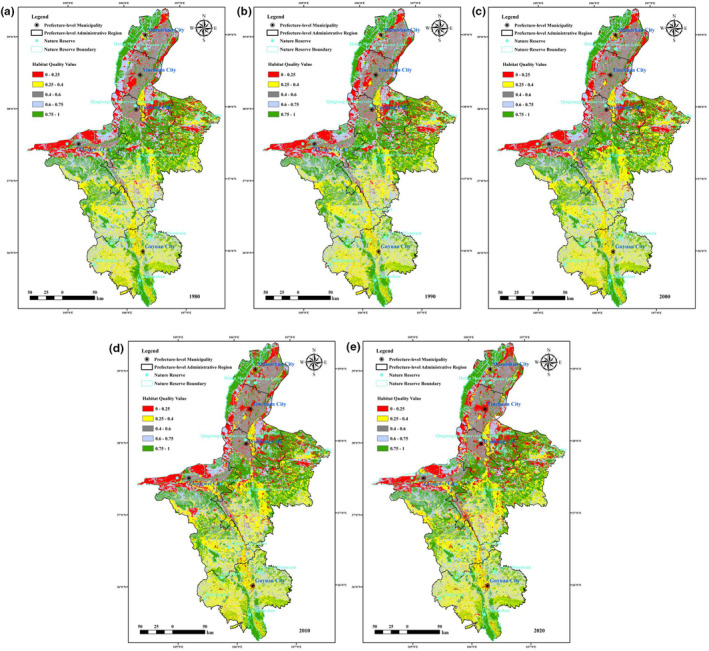
Spatial distribution of habitat quality in Ningxia from 1980 to 2020.

**FIGURE 4:**
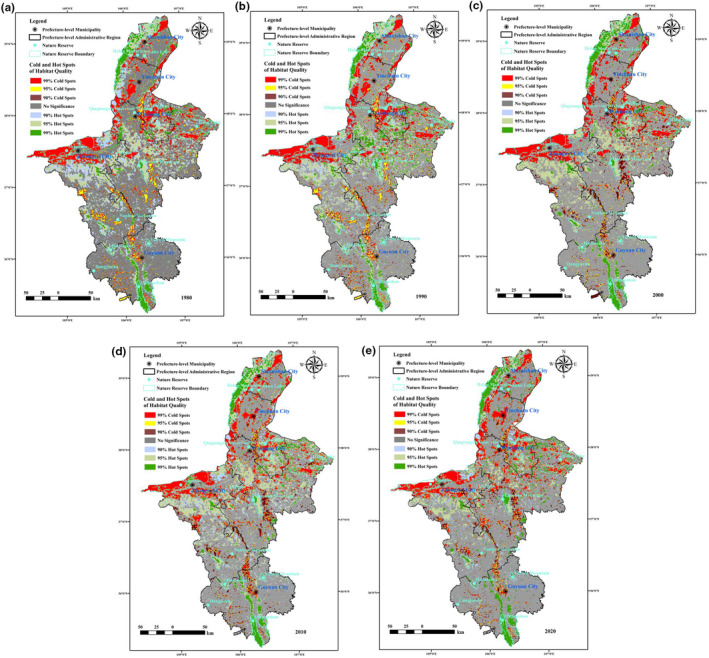
Cold and hot spot analyses of habitat quality in Ningxia from 1980 to 2020.

**FIGURE 5:**
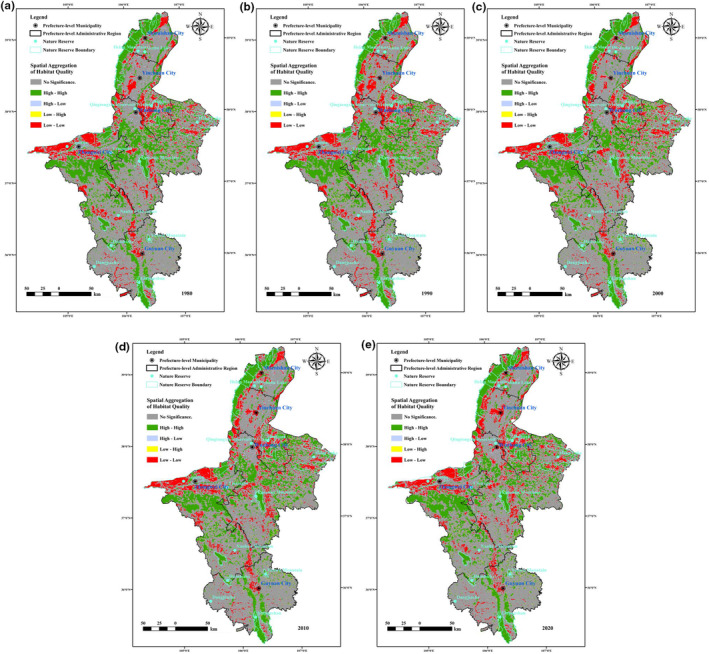
Spatial autocorrelation analysis of habitat quality in Ningxia from 1980 to 2020.

In 1980, 1990, 2000, 2010 and 2020, the global Moran's index of habitat quality was 0.48, 0.49, 0.47, 0.46 and 0.46, respectively. The values of the corresponding *p* and *Z* scores were much less than 0.05, greater than 2.58, indicating that there was a significant correlation. In terms of spatial distribution (Figures 3a‐e and 4a‐e), the areas with high habitat quality determined with a 99% confidence level (5.12%–7.13%) were mainly distributed in the Helan Mountains in northern Ningxia, the Liupan Mountains in the south and nature reserves such as the Bainingtan and Niushu Mountains in central Ningxia. The areas with very high values determined with a 95% confidence level (11.43%–12.12%) were distributed in the areas around Xiangshan Mountain in the southwest and Luoshan Mountain and Haba Lake in the central part of the region. The hotspots (identified with a 90% confidence level) (4.72%–8.61%) were scattered throughout the above areas. The coldspots (identified with a 99% confidence level) (9.41%–10.25%) were mainly distributed along the edge of the Helan Mountains and the western Shapotou area. In addition, the areas with dense human construction land were essentially coldspots (identified with a 99% confidence level). The coldspots identified with 95% (2.59%–4.28%) and 90% (3.36%–4.73%) confidence levels were smaller in size, mainly located in the central arid area and interspersed with the hotspots. In general, the hotspots of habitat quality in Ningxia were mainly located in the north and south and in the central areas where nature reserves have been established. The coldspots were mainly located in the west and in areas where cultivated land is used more frequently, and the area of non‐significant areas was close to half of the total area (55.84%–60.21%) of Ningxia, exhibiting a more random distribution. The results of the spatial autocorrelation analysis (Figure 5a‐e) showed that there were mainly two aggregation patterns of high–high and low–low aggregation in habitat quality in Ningxia. The areas with high habitat quality showed significantly high–high aggregation (22.02%–24.16%), and the distribution range was more consistent with that of the habitat quality hotspots. The low habitat‐quality areas exhibited significantly low–low aggregation (12.18%–13.13%), which was basically consistent with the distribution of habitat‐quality coldspots. The areas of high–low aggregation (2.33%–2.98%) and low–high aggregation (0.86%–1.28%) were smaller in scope, and the areas that did not exhibit significant correlation (59.85%–61.46%) with the geographical environment occupied half of the total area of Ningxia. The results indicated that the high and low values of habitat quality in Ningxia were significantly influenced by geospatial factors and had strong spatial aggregation.

#### 3.2.2 Spatial–temporal change characteristics

**FIGURE 6:**
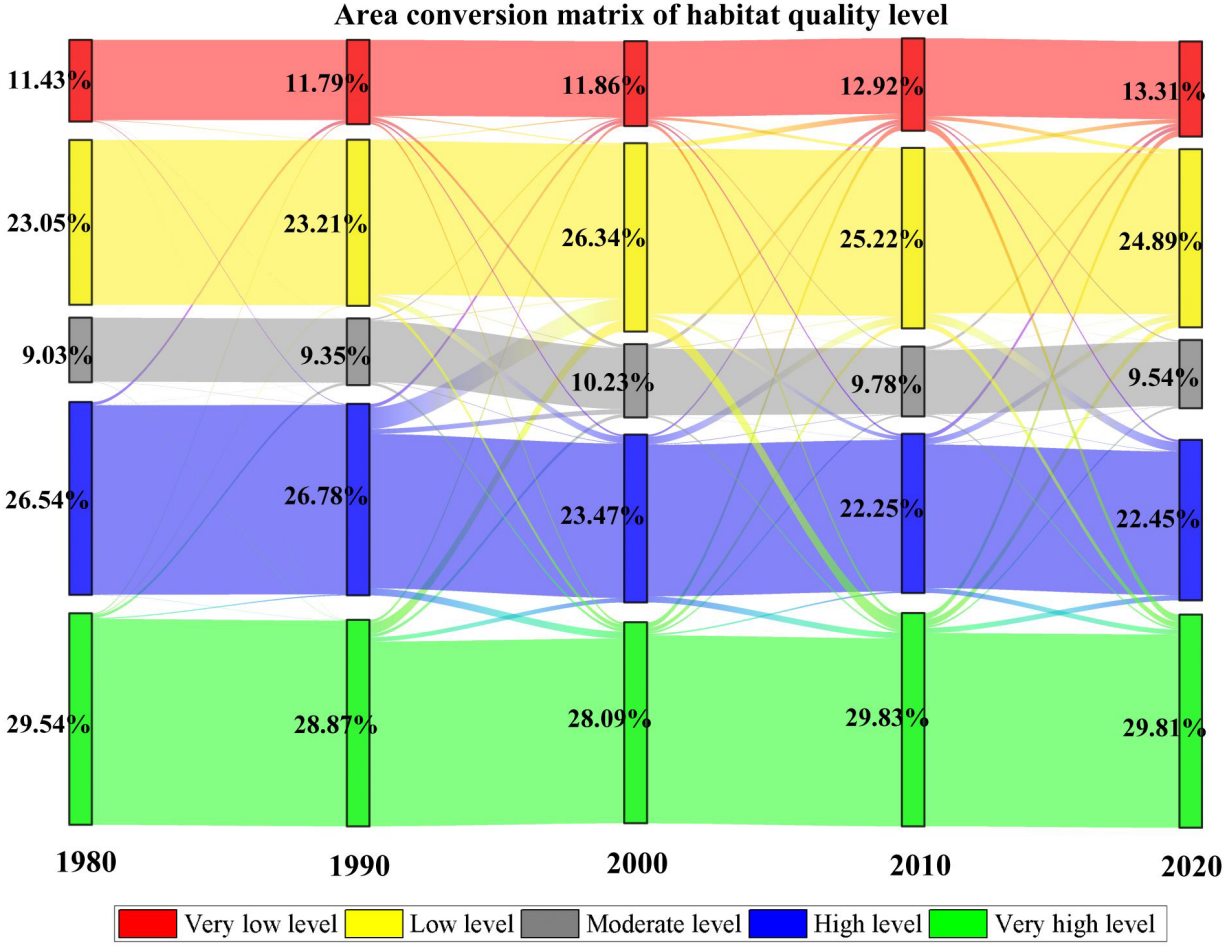
Habitat quality transfer matrix from 1980 to 2020.

The average habitat quality in Ningxia showed an increasing trend from 1980 to 1990 (0.0045), a decreasing trend from 1990 to 2020 and an overall decreasing trend over the 40 years, with less fluctuation (−0.0217). From 1980 to 2020, the overall proportion of very low, low and moderate habitat quality levels in Ningxia (1.8867%, 1.8378% and 0.5140%, respectively) increased, and the proportion of high habitat quality also increased (0.2670%) and only the proportion of very high habitat quality decreased (−4.5055%). In different periods, the average habitat quality in Ningxia decreased the most, and the proportion of different levels of habitat quality changed the most from 1980 to 2000. From 2000 to 2020, although areas with very high habitat quality continued to change to very low and high levels, the rate of change gradually slowed. Overall, the trend of habitat quality change in Ningxia during the 40 years was from very high habitat quality levels to moderate, low, very low and high levels (Figure 6).

**FIGURE 7:**
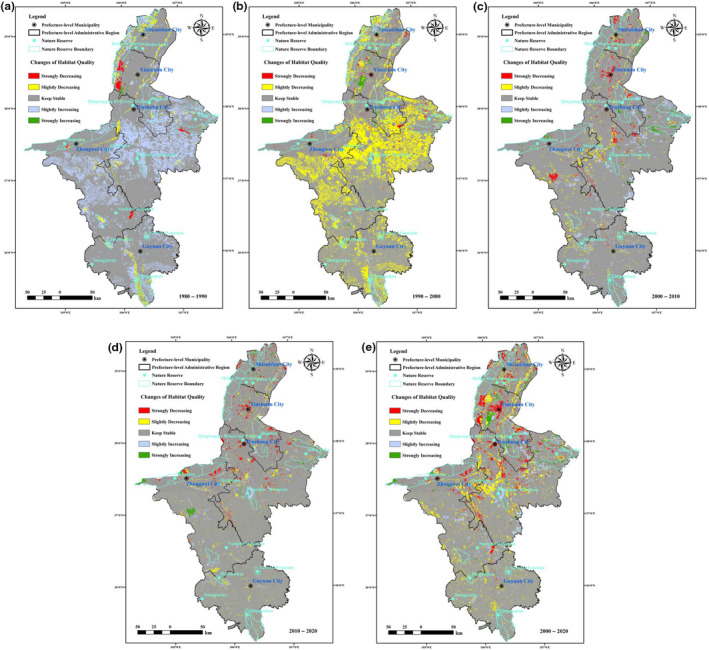
Changes of habitat quality in Ningxia in various periods from 1980 to 2020.

As shown in Figure 7a‐e, the habitat quality changes in Ningxia in the five time periods (1980–1990, 1990–2000, 2000–2010, 2010–2020 and 1980–2020) were classified into five levels: drastic decrease, slight decrease, stable, slight increase and drastic increase. From the spatial variation of each period, the habitat quality of Ningxia exhibited a decreasing trend in almost all areas from 1980 to 1990 and a decreasing trend in most areas from 1990 to 2000, except for some areas in the north. In the period from 2000 to 2010, some areas in eastern and southern Ningxia showed an obvious improvement in habitat quality; at the same time, the number of patches exhibiting a sharp decrease began to increase. In the period from 2010 to 2020, the areas with decreasing and increasing habitat quality in Ningxia were almost the same. The habitat quality decreased in some areas in the central and northern parts, and the habitat quality gradually improved in some patches in the southern and eastern parts. Overall, the habitat quality of Ningxia fluctuated greatly from 1980 to 2000, especially in approximately 1990, when the habitat quality in the central region was obviously improving. From 2000 to 2020, the habitat quality of Ningxia remained stable or slightly decreasing, and the areas of high and very high levels gradually increased in some areas of Wuzhong and Zhongwei cities in the central region; the areas of very low and low levels in some areas in the fringes also increased. From 1980 to 2020, the habitat quality of Ningxia remained stable in general; the patches with slight declines and sharp increases were widely distributed, while the patches with sharp declines and slight increases were relatively concentrated. The areas where habitat quality decreased sharply were mainly located in the southern foothills of the Helan Mountains and the densely cultivated areas in the central part of the country, while the areas where habitat quality increased sharply were concentrated in the north and west.

### 3.3 Response of habitat quality to land use and landscape pattern changes

#### 3.3.1 Response of habitat quality to land use changes

In terms of the average habitat quality of different land use types, in 2020, the habitat quality of forestland was the highest, and the order of the other types was as follows: grassland > water > cultivated land > untapped land > construction land. This result indicated that patches with good ecological environments usually had higher habitat quality, and the habitat quality of human land, such as cropland and construction land, was usually lower. The habitat quality of cropland was higher than that of construction land, indicating that cropland has a certain habitat maintenance function, although it is more disturbed by humans (Table 6).

**TABLE 6:** Habitat quality of different land use types in Ningxia from 1980 to 2020.

Land use category	Cultivated land	Forest land	Grassland	Water area	Construction land	Untapped land
1980	0.4514	0.9982	0.7747	0.8644	0.0035	0.0105
1990	0.4516	0.9967	0.786	0.7467	0.0041	0.0136
2000	0.4654	0.9440	0.7237	0.7087	0.0619	0.0785
2010	0.4727	0.9255	0.7086	0.6765	0.0848	0.1410
2020	0.4634	0.9033	0.7022	0.6663	0.0824	0.1549

Using ArcGIS partition statistics for the table function, the average habitat quality of different land use types in Ningxia from 1980 to 2020 was determined, and the amount of change was calculated. The results showed that the average habitat quality of cultivated land (0.0120), construction land (0.0789) and untapped land (0.1444) in Ningxia increased from 1980 to 2020, whereas the average habitat quality of forestland (−0.09488), grassland (−0.0725) and water (−0.19811) decreased (−0.0949), grassland (−0.0725) and watershed (−0.1981) had lower mean habitat quality. Habitat quality did not vary significantly across time for different land use types, but the average habitat quality exhibited a decreasing trend for all land use types except for untapped land during the last decade (from 2010 to 2020).

A total of 275 sample sites exhibited deteriorating habitat quality, 1593 sample sites showed stable habitat quality and 184 sample sites exhibited increasing habitat quality. Forty years later, the land use types with decreasing habitat quality were primarily construction land, untapped land and cultivated land, whereas the land use types with increasing habitat quality were mostly forestland, grassland and water, and included a small amount of cultivated land. The areas with stable habitat quality were more complex and mainly included areas with unchanged land use or changes in land use characterized by small decreases or increases (Table 7). In general, a change in land use to forestland, grassland and water is favourable for improving habitat quality, while a change to cultivated land, construction land and untapped land leads to a decrease in habitat quality. However, habitat quality changes are not only related to land use changes but also to the factors of patch size, density, shape and connectivity, so it is especially important to consider the effects of these factors as well.

**TABLE 7:** Habitat quality changes caused by land use transfer in Ningxia from 1980 to 2020.

Type of land change	Amount of habitat quality change	Type of land change	Amount of habitat quality change	Type of land change	Amount of habitat quality change	Type of land change	Amount of habitat quality change
2→5	−0.958	4→1	−0.380	3→3	−0.001	5→1	0.429
2→6	−0.921	3→1	−0.307	1→1	0.001	6→1	0.467
4→6	−0.904	2→3	−0.157	6→6	0.002	1→2	0.535
2→6	−0.841	4→3	−0.109	5→5	0.004	5→3	0.624
3→5	−0.753	2→4	−0.055	4→2	0.099	6→3	0.721
3→6	−0.728	4→4	−0.045	3→4	0.134	5→4	0.772
1→5	−0.493	5→6	−0.031	3→2	0.203	5→2	0.784
2→1	−0.474	6→5	−0.005	1→3	0.323	6→2	0.934
1→6	−0.411	2→2	−0.003	1→4	0.360	6→4	0.938

#### 3.3.2 Response of habitat quality to landscape pattern changes

**FIGURE 8:**
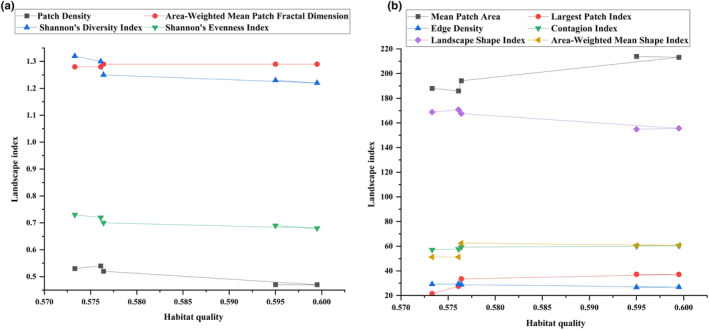
Relationship between habitat quality and landscape pattern index in Ningxia.

As seen in Table 4 and Figure 8a,b, from 1980 to 2020, as the SHDI, SHEI, PD, LSI and ED increased, the habitat quality of Ningxia decreased; the overall landscape level of Ningxia showed continuous fragmentation, the frequency of small patches increased year by year, the connectivity and connectivity level of the landscape decreased year by year, and it was more difficult to connect landscape between patches within the landscape. The richness of the landscape and patch types facilitates species habitat selection, but complexity and diversity can reduce the stability of species survival at the habitat level. AWMPFD, MPA, CONTAG, AWMSI, LPI and habitat quality generally remained in proportion to each other, indicating that a more complete, larger and well‐connected landscape pattern is more conducive to improving habitat quality.

**FIGURE 9:**
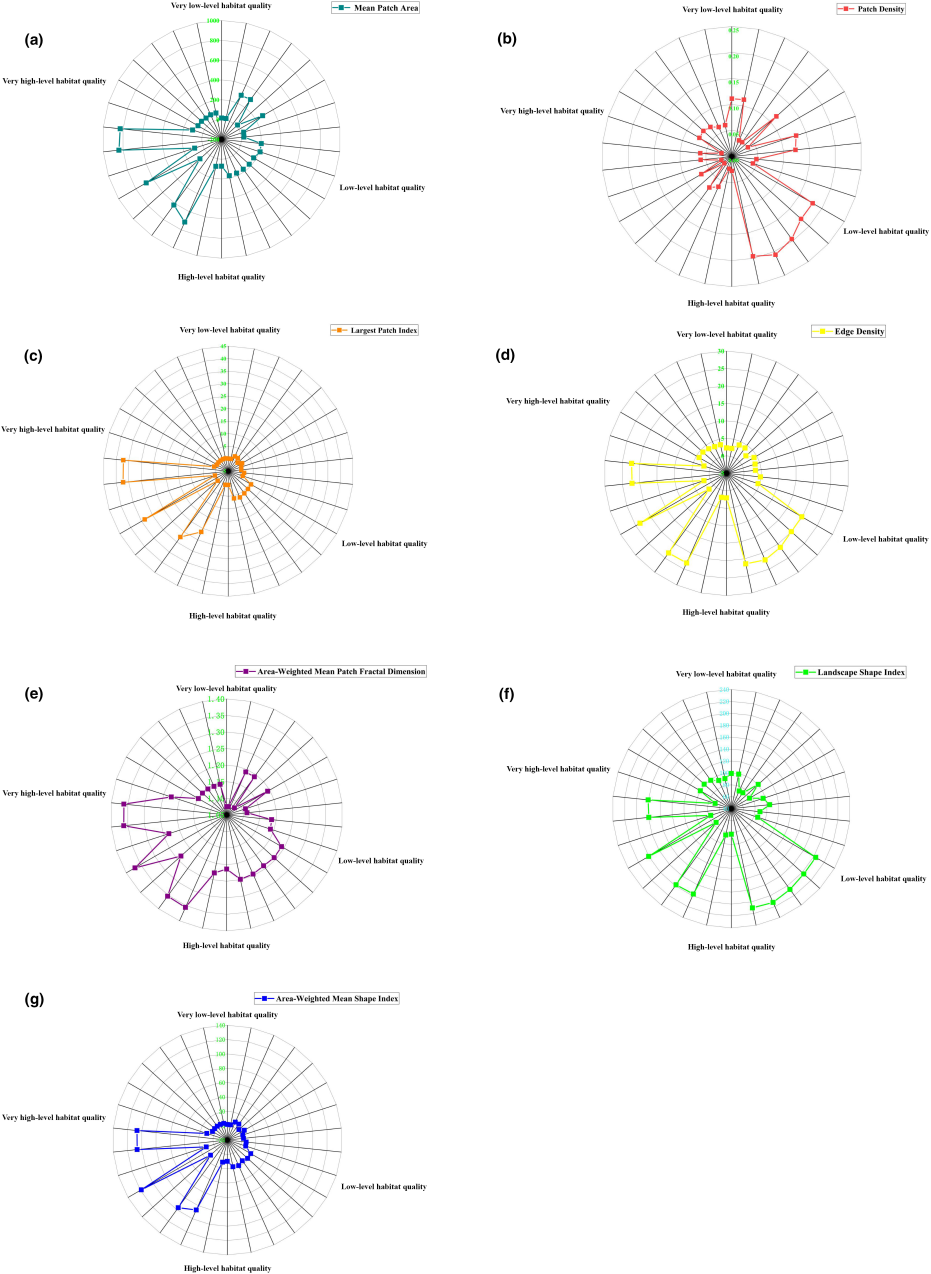
Response of habitat quality to landscape pattern index based on different land types in Ningxia.

The size of landscape pattern indices for different land use types in Ningxia from 1980 to 2020 was counted using the ArcGIS zone‐to‐table function for seven indices: PD, LPI, ED, LSI, MPA, AWMSI and AWMPFD. Radar plots were generated with the corresponding habitat quality. As indicated by the results in Figure 9a–g, the size of LPI, AWMFPD, AWMSI, MPA and habitat quality were roughly positively correlated, that is, the habitat quality was generally greater when these indices increased. The size of PD and habitat quality were roughly inversely correlated, that is, the habitat quality was generally lower when these indices increased. The positive and negative correlations between LSI and ED and habitat quality were not obvious, and the changes in habitat quality with the changes in these indices were uncertain. In general, habitat fragmentation and complexity usually reduce habitat quality, but the effect is not clear, and the threshold of abrupt change is difficult to determine.

## 4 DISCUSSION

### 4.1 Adaptability analysis of research methods

In terms of research methodology, this study adopts the Invest model, which is a commonly used habitat quality assessment model in international and academic circles, and the accuracy of the methodology is guaranteed. However, it is worth noting that the basic data used in the habitat quality assessment module of the Invest model are mainly land use data as well as setting the degree of habitat threat and sensitivity of each land use type based on surveys and studies. Therefore, this study had to encounter several troubling problems, firstly, the higher the precision of land use classification is more friendly the results, however, this study used the 30‐m resolution land classification system of the Chinese Academy of Sciences (CAS), which has a large room for improvement in the fineness of land use resolution and categories. In terms of parameter setting, many studies have focused on the impact of human road construction on pristine habitats, but we generally categorized them as construction land, which is also worth improving; secondly, the Invest model is mostly applicable to the assessment of habitat quality at the macro scale, so it lacks the assessment of specific species and habitats at the micro‐scale. As we know, the survival, reproduction and dispersal of biological populations, especially endangered populations, are extremely dependent on the ecological factors necessary to adapt to the habitat, which can be temperature, water, space, food, etc., and the specific behaviour of human beings in dealing with nature can also cause interference with the habitat, so the present study of the habitat quality assessment is only suitable for the analysis of the quality of habitats on a larger spatial scale in Ningxia and lacks the tracking of microhabitats. The present assessment of habitat quality is only suitable for the analysis of habitat quality at larger spatial scales in Ningxia and lacks tracking of tiny habitats. Finally, in searching for the causes of habitat quality changes, we only linked habitat quality to changes in land use and landscape patterns and lacked the coupling of specific indicators related to socioeconomics and physical geography to analyse the drivers of habitat quality in Ningxia, while failing to use a more accurate analytical method to identify the main drivers that cause spatial and temporal distributions of, and changes in, Ningxia's habitat quality, and, synthesizing the methodological limitations of the present study, in future, we will further study the changes in Ningxia habitat quality and its driving mechanisms from the following aspects: (1) On the basis of the Invest model habitat assessment, identify the key areas for habitat protection in Ningxia, such as nature reserves and human‐intensive zones and comprehensively utilize the traditional biohabitat survey and analysis method, maxent ecological niche model and other methods to collect the ecological factors required for a specific species under a specific habitat, so as to analyse the living conditions and habitat suitability of the populations; (2) Fully consider the human activity factors and natural background conditions of the habitat quality of the image biological populations, screen the applicable driver evaluation index system and search for the key factors affecting the changes of the habitat quality in Ningxia based on the more accurate methods such as geographically weighted regression, geographic detector, image‐by‐image meta‐regression, etc. and determine the specific countermeasures and suggestions for the conservation of biological diversity in Ningxia in combination with key drivers in the sub‐region. We will also identify specific countermeasures and suggestions for biodiversity conservation in Ningxia in combination with the key drivers in the sub‐region.

### 4.2 Habitat quality characteristics of Ningxia

Habitat quality in Ningxia was generally moderate, consistent with the characteristics of an ecologically fragile area. At the same time, there was a tendency for vegetation area to increase, which was similar to the findings of Bao (2022) and He et al. (2020). The habitat quality in Ningxia has fluctuated between improving and worsening during the 40 years, which is related to the conversion of grassland to cultivated land and construction land and some cultivated land to grassland and woodland. Landscape fragmentation increased and the average patch size and connectivity decreased, which led to the expansion of low habitat quality. In the InVEST model, habitat quality size was mainly related to two factors: whether the land type acted as a habitat threat source and the sensitivity of the land type to the threat source. Powers and Jetz (2019), Crooks et al. (2017) and Hautier et al. (2015) argued that urban expansion, deforestation, land conversion, and road construction caused by human development activities lead to the destruction of original habitats, increased landscape fragmentation and habitat incompleteness, which will result in reduced habitat suitability and habitat degradation, and habitat quality in these areas will become extremely low. The main ecosystem types in Ningxia were grassland and cultivated land, and changes determined the transformation of ecological threat sources, while the increase in construction land led to the expansion of ecological threat sources. The closer an area is to the threat source, the lower the habitat quality performance. In addition, human purposeful construction and development activities and the extension of road systems change the form of the sensitivity of habitat threats from linear to exponential. The proportion of areas with poor habitat quality in Ningxia was large and tended to decrease over time, but with the implementation of relevant ecological protection measures, the proportion of land with high and very high levels of habitat quality increased significantly from 2000 to 2020. In approximately 2000, Ningxia responded to the national call to implement major projects, such as returning farmland to forests and grasslands, and during the 20 years leading up to 2021, Ningxia completed the conversion of 13.45 million Mu (Mu, Chinese Unit of land measurement, i.e. commonly 666.7m^2^), which significantly improved the ecological environment. However, due to urbanization and the increase in food production on cultivated land, the habitat quality in Ningxia slightly declined from 2000 to 2020 and did not improve significantly, probably due to the frequent conversion of cultivated land‐forestry‐grassland‐construction land in Ningxia. The landscape ecosystems on which habitat quality depends were damaged and lost to different degrees. Frequent land use caused habitat degradation, which can be seen from the map of habitat quality degradation distribution in Ningxia.

Based on the results of the landscape pattern index from 1980 to 2020, the development of Ningxia landscape types exhibited a trend toward complexity, diversification and enrichment, which, on the one hand, is conducive to increasing species habitat types and active population exchange, but, at the same time, can lead to habitat fragmentation and isolation, which is not conducive to species survival. The complexity of landscape patterns is closely related to human disturbance, especially land conversion, environmental pollution, and climate change, which accelerate the loss and quality of biological habitats, thus causing biodiversity to decline (Beketov et al., 2013; Mantyka‐Pringle et al., 2012; Newbold, 2018). Intact and larger habitats are obviously conducive to the survival and reproduction of organisms. In contrast, the more fragmented and dense the patches within the landscape are, the less conducive to the exchange of populations. On the other hand, the greater the spread of the landscape is, the more ecological corridors there are, and the more homogeneous the landscape material information and energy are, the more favourable the stability and mutual dispersal of biological populations are. Based on the present research results, determining the thresholds of habitat quality increase and decrease caused by habitat patch area, density, edge and shape is an important topic of research for the future.

Habitat quality is closely related to land use and increasing vegetation cover and establishing protected areas are important means of maintaining species habitat (Arroyo‐Rodríguez et al., 2020; Nagendra et al., 2013). Improving species suitability first requires maintaining the living environment for species survival. Based on the results of the study, the protection of habitat quality first requires controlling the frequent disturbance of anthropogenic activities and reducing the generation of ecological threat sources, paying special attention to the invisible road network and other exponential factors that are detrimental to the habitat. The Ningxia reforestation and grassland restoration project helped greatly to improve habitat quality. Therefore, when funds, measures, and local residents are available, it is important to make full use of ecological protection projects to restore damaged habitats while protecting the original habitats. Ningxia established a multilayered and multilevel nature reserve system at the provincial, municipal and county levels, and the importance of habitat protection and habitat quality will be further enhanced in the context of optimizing the spatial protection and development pattern of the country.

### 4.3 Conservation of biodiversity in ecologically fragile areas

Ningxia is located in the arid and semiarid region in western China in the agricultural and pastoral interlacing zone, with a comprehensive range of ecosystem types but relatively harsh climatic and geographical conditions. Vegetation types include scrub, grassland, meadow, sand vegetation and desert vegetation. The grassland types are mainly desert grassland and dry grassland, and the regional ecological environment is extremely sensitive to changes and disturbances in the external environment. Due to unreasonable exploitation of land resources, insufficient awareness of biodiversity, overuse of biological resources and unfavourable conservation measures, degradation of natural grasslands, imbalance of forest systems and destruction of the structure and function of agro‐ecosystems have occurred. As a result, the ecosystems in Ningxia have become unbalanced, extremely unstable and vulnerable to natural risks, which has become a bottleneck limiting regional socioeconomic development. Therefore, the conservation of genetic, species, community, ecosystem and landscape diversity is essential to maintain Ningxia's fragile ecological environment. The interlocking agricultural and livestock areas in northern China are coupled with traditional agricultural and livestock areas, which are extremely sensitive to external environmental changes and disturbances; these are the areas that are most profoundly and frequently affected by external disturbances. The future study of habitat quality from the perspectives of ecosystem conservation, population income enhancement and sustainable development will help Ningxia achieve harmonious development between humans and nature and provide a scientific reference for biodiversity conservation, ecological security, ecological environmental protection and sustainable development of its ecosystem in the study area and other similar ecologically fragile areas.

Overall, Ningxia has a poor natural background, especially in arid and semi‐arid areas, where rainfall is scarce, ecosystem structure is simple, biodiversity is homogeneous, and habitat conservation is extremely important for maintaining ecosystem stability. This study shows that most of the areas with better habitat quality in Ningxia are located in areas with better natural background, such as forest ecosystems and grassland ecosystems, and the urban ecosystems that suffer the most intense human interference do not have any habitat suitability for all, whereas the cultivated ecosystems are more special, on the one hand, the intensity of cultivated land use, such as the higher rate of pesticide and chemical fertilizer application, the more detrimental to the survival of native species, but at the same time, due to the implementation of a series of human But at the same time, due to the implementation of a series of human ecological protection measures such as soil and water conservation, which to a certain extent protects the structural and functional integrity of the ecosystem, which is conducive to the survival and reproduction of organisms. From the point of view of land use and landscape pattern, the more drastic the land use change, the more fragmented the landscape, the lower the quality of the habitat, the intact and suitable habitat size is the basic element of habitat maintenance, in addition to changes in the conditions of food, space and other conditions within the landscape, the habitat suitability also increases. Therefore, based on the fragile ecological environment and poor socioeconomic development level in Ningxia and ecologically fragile areas, we propose the following strategies to better promote local biodiversity conservation: (1) Maintain the structural and functional integrity of the ecosystems as much as possible, especially for areas of high biodiversity, such as pristine forests, meadows, wetlands, etc., and reduce the disturbance of human activities such as mineral excavation and deforestation, etc.; (2) Adopt ecological protection measures to improve the quality of the habitat, such as the establishment of nature reserves based on the protection of local biological populations, to reasonably and effectively maintain the diversity and stability of the habitat and ecological factors, and at the same time, increase the investment of personnel and funds, implement a series of ecological protection projects to improve the ecosystem's function of soil and water conservation, windbreaks and sand fixation, and water containment, so as to make the ecosystem functionally sound. (3) Finally, to determine the key factors affecting the quality of the habitat, coupled with the synergistic development of society and nature, to increase ecological compensation, the rational and effective use of ecological migration, ecological payment transfer and other policies to enhance the well‐being of local residents, so as to achieve harmonious coexistence between residents and nature, and to stimulate the enthusiasm of the local residents of the protection of biodiversity.

## 5 CONCLUSION

The conclusions of this paper are as follows: (1) From 1980 to 2020, the largest changes in the proportion of land use area in Ningxia were in construction land and cultivated land, which increased by 2.79% and 2.74%, respectively, while the area of grassland decreased by 5.36%. The proportion of increased forest land area (0.96%) and decreased water area (−0.29%) and untapped land (−0.83%) is relatively low. The MPA (), LPI, CONTAG and AWMSI of Ningxia landscape types exhibited a decreasing trend. PD, ED, SHDI and SHEI showed an increasing trend and AWMFPD basically remained unchanged. The LSI exhibited an increasing and then decreasing trend and the landscape pattern obviously tended to be fragmented and complicated. (2) From 1980 to 2020, the average level of habitat quality in the Ningxia region was 0.5950, 0.5995, 0.5764, 0.5761 and 0.5733, and the habitat quality of the ecological environment was generally moderate over the past 40 years but exhibited a fluctuating downward trend. The spatial distribution of habitat quality throughout Ningxia varied significantly, and there was a significant aggregation of coldspots and hotspots. Hotspots (25%) were mainly found in the north and south as well as in areas such as nature reserves, showing significantly high–high aggregation, whereas coldspots (13%) were mainly located in the west and in areas where the land use is mainly cultivated land, showing significantly low–low aggregation. (3) The MPA, LPI, CONTAG and AWMSI of Ningxia landscape types exhibited a decreasing trend. PD, ED, SHDI and SHEI showed an increasing trend and AWMPFD basically remained unchanged. The LSI exhibited an increasing and then decreasing trend and the landscape pattern obviously tended to be fragmented and complicated. The analysis of habitat quality changes caused by land use and landscape patterns showed that forestland, grassland and watersheds were favourable for maintaining habitat quality, while the habitat quality of cropland, construction land and untapped land was usually low. There was uncertainty regarding the threshold of low and high mutation of habitat quality. Landscape patterns also affected habitat quality and unfavourable factors such as smaller habitat patch size, higher density, complex shape and reduced connectivity within the landscape resulted in declines in habitat quality.

## AUTHOR CONTRIBUTIONS

**Ding Wang:** Conceptualization (lead); data curation (lead); formal analysis (lead); funding acquisition (supporting); investigation (lead); methodology (lead); project administration (equal); resources (equal); software (lead); supervision (equal); validation (equal); visualization (lead); writing – original draft (lead); writing – review and editing (equal). **Haiguang Hao:** Conceptualization (lead); data curation (equal); formal analysis (equal); funding acquisition (lead); investigation (equal); methodology (equal); project administration (lead); resources (lead); software (equal); supervision (equal); validation (equal); writing – original draft (equal); writing – review and editing (equal). **Lihui Sun:** Conceptualization (supporting); data curation (supporting); formal analysis (equal); funding acquisition (equal); investigation (equal); methodology (supporting); project administration (supporting); resources (equal); software (equal); supervision (supporting); validation (supporting); visualization (supporting); writing – original draft (equal); writing – review and editing (equal). **Hao Liu:** Conceptualization (supporting); data curation (supporting); formal analysis (supporting); funding acquisition (supporting); investigation (equal); methodology (supporting); project administration (equal); resources (supporting); software (supporting); supervision (equal); validation (supporting); visualization (equal); writing – original draft (equal); writing – review and editing (equal). **Yuyang Li:** Conceptualization (equal); data curation (supporting); formal analysis (supporting); funding acquisition (supporting); investigation (supporting); methodology (equal); project administration (supporting); resources (supporting); software (supporting); supervision (supporting); validation (supporting); visualization (supporting); writing – original draft (equal); writing – review and editing (equal).

## FUNDING INFORMATION

This research was funded by the National Natural Science Foundation of China (grant no. 41871196) ＆ National philosophy and Social Science Foundation of China (grant no. 18ZDA048).

## CONFLICT OF INTEREST STATEMENT

The authors declare no conflicts of interest.

## Data Availability

The data and materials used in the text were obtained from legitimate sources or official citations, and no related misappropriation or other undesirable practices were involved. All data and material sources are described in the text, and the authors can provide additional information upon request. All data generated in the text have been included within the article for easy reference or citation.
